# Addition of bevacizumab enhances antitumor activity of erlotinib against non-small cell lung cancer xenografts depending on VEGF expression

**DOI:** 10.1007/s00280-014-2610-x

**Published:** 2014-10-26

**Authors:** Heyan Li, Koichi Takayama, Shuo Wang, Yoshimasa Shiraishi, Keisuke Gotanda, Taishi Harada, Kazuto Furuyama, Eiji Iwama, Ichiro Ieiri, Isamu Okamoto, Yoichi Nakanishi

**Affiliations:** 1 Research Institute for Diseases of the Chest, Graduate School of Medical Sciences, Kyushu University, 3-1-1 Maidashi, Higashiku, Fukuoka, 812-8582 Japan; 2 Department of Clinical Pharmacokinetics, Graduate School of Pharmaceutical Sciences, Kyushu University, 3-1-1 Maidashi, Higashiku, Fukuoka, 812-8582 Japan

**Keywords:** Bevacizumab, Drug concentration, Erlotinib, Non-small cell lung cancer, VEGF protein

## Abstract

**Purpose:**

Erlotinib, an epidermal growth factor receptor (EGFR) tyrosine kinase inhibitor (TKI), and bevacizumab, an anti-vascular endothelial growth factor (VEGF) agent, are promising therapies for advanced non-small cell lung cancer (NSCLC). Our study was aimed to determine whether there were conditions under which the addition of bevacizumab would enhance the antitumor activity of erlotinib against NSCLC tumors in vitro and in vivo.

**Methods:**

MTS was for NSCLC cell (PC9, 11–18, H1975, H157, H460 and A549) growth assay in vitro. ELISA was for VEGF protein assay in cells and tumor tissues. Mouse xenograft models were established with H157, H460 and A549 with primary resistance to erlotinib and treated with erlotinib plus bevacizumab or each agent alone. Erlotinib concentrations in tumors were determined by high-performance liquid chromatography.

**Results:**

Bevacizumab alone did not inhibit NSCLC cell growth in vitro. In primarily erlotinib-resistant NSCLC cells, the levels of VEGF protein were highest in H157 cell followed in order by H460 and A549 cells. In vivo, bevacizumab alone significantly inhibited tumor growth only in xenograft models with high (H157) and/or moderate (H460) levels of VEGF protein. A combination of erlotinib and bevacizumab partially reversed resistance to erlotinib in H157 xenografts (high VEGF level) with increasing intratumoral erlotinib concentrations, but not in H460 (moderate) or A549 (low) xenografts.

**Conclusions:**

These results support that combined with anti-VEGF therapy could enhance antitumor activity of anti-EGFR therapy and/or partially reverse resistance to EGFR TKI, by increasing EGFR TKI concentration in specific tumors that express high levels of VEGF protein.

**Electronic supplementary material:**

The online version of this article (doi:10.1007/s00280-014-2610-x) contains supplementary material, which is available to authorized users.

## Introduction

Over the past 10 years, targeted therapies, such as small molecule inhibitors and monoclonal antibodies, have improved the treatment of cancers [1]. Lung cancer is the leading cause of cancer-related deaths worldwide, and approximately 75 % of patients with non-small cell lung cancer (NSCLC, >85 % of lung cancer) present with advanced stage disease, which is unresectable or metastatic [2, 3]. Thus, the epidermal growth factor receptor (EGFR) tyrosine kinase inhibitors (TKIs), erlotinib and gefitinib, and the monoclonal antibody against human vascular endothelial growth factor (VEGF), bevacizumab, are now components of treatment regimens for advanced NSCLC [4]. These targeted drugs received approval by the United States Food and Drug Administration (FDA) for the treatment of patients with advanced or metastatic NSCLC, respectively [5–7].

Recently, combination therapy has received much attention because of its potential to reduce resistance to targeted therapies, and/or improve efficacy through the inhibition of multiple receptors. At the molecular level, erlotinib and bevacizumab target different pathways (EGFR and VEGF), which share both parallel and reciprocal downstream signaling mechanisms [8, 9]. Phase 1/2 trials demonstrated that median overall survival (OS) was better after treatment with erlotinib plus bevacizumab than with bevacizumab plus chemotherapy or chemotherapy alone in patients with relapsed and refractory non-squamous NSCLC [10, 11]. However, phase III trials showed the addition of bevacizumab to erlotinib improved progression-free survival (PFS) but not OS in patients with recurrent, advanced or metastatic NSCLC [12, 13]. The reason for the lack of additive benefits to erlotinib plus bevacizumab has remained speculative.

Since an overactive VEGF pathway independent of EGFR plays a role in resistance to EGFR TKI [14], dual inhibition of both pathways may prevent resistance through VEGF [9]. In addition, several studies have focused on the effect of bevacizumab on drug delivery to tumors [15–17]. These studies may lead to a breakthrough that explains the reason for the lack of synergy in preclinical studies and clinical trials. In our study, we hypothesized that bevacizumab affects the antitumor activity of erlotinib and the available concentration of erlotinib in vivo depending on the levels of VEGF expression in NSCLC cells. We examined the relationship between the efficacy of bevacizumab and the levels of VEGF protein in NSCLC cells. Then we investigated antitumor activity of erlotinib plus bevacizumab in erlotinib-resistant NSCLC xenograft models and evaluated the levels of erlotinib in tumor tissues.

## Materials and methods

### Cell cultures and reagents

The human NSCLC cell lines PC9, 11–18, H1975, H157, H460, A549 and the normal human bronchial epithelial cell line BEAS-2B were obtained from the American Type Culture Collection. Cells were cultured in RPMI 1640 medium (Gibco, Carlsbad, CA) or DMEM/F-12 medium (Gibco, Carlsbad, CA) supplemented with 10 % fetal bovine serum and 1 % penicillin–streptomycin at 37 °C in 5 % carbon dioxide. Bevacizumab and erlotinib were provided by Chugai Pharmaceutical Co. Ltd (Tokyo, Japan) and Cayman Chemical (Ann Arbor, MI), respectively.

### Cell growth assay

Six NSCLC cell lines were seeded (~2,000–5,000 cells per well, depending on cell type) onto 96-well plates. After 24 h of incubation, cells were treated with erlotinib (0–20 µmol/L), bevacizumab (0–20 ng/mL) or a combination of these agents (erlotinib 1 µmol/L; bevacizumab 10 ng/mL) for 72 h in serum-containing medium. The viability was determined by MTS assay (Promega, Madison, WI) according to the manufacturer’s instructions.

### Human VEGF ELISA assay

The methods of VEGF quantification have been described previously [18, 19]. Briefly, the supernatant of cell culture media and homogenized tumor samples were collected for the assays. The human VEGF protein was determined with Quantikine ELISA kit (R&D Systems, Minneapolis, MN), according to the manufacturer’s instructions. Total protein levels were quantified by BCA assay (Thermo Scientific, Rockford, IL).

### Xenograft models

Female BALB/cAJcI-nu/nu mice (5- to 6-week old) were obtained from CLEA Japan, Inc (Tokyo, Japan). Mice were kept in a 12-h light and dark cycle, and acclimatized for 1 week before the study. The experimental protocols were reviewed and approved by the Kyushu University Animal Care and Use Committee (Fukuoka, Japan). “Principles of laboratory animal care” (NIH publication No. 85-23, revised 1985) were followed or comply with standards equivalent to the UKCCCR guidelines for the welfare of animals in experimental neoplasia [20].

Human NSCLC cells (5–10 × 10^6^ H157, H460 or A4549 cells/mouse) were injected subcutaneously into the mice. When established tumors became palpable (~100–300 mm^3^), mice were randomized into control and treatment groups and treated with vehicle, bevacizumab (5 mg/kg/twice weekly, i.p.), erlotinib (100 mg/kg/day, gavage) or erlotinib plus bevacizumab for the indicated periods. Moribund animals were killed to reduce suffering. Tumor volume and body weight were measured twice weekly. Tumor volume equation is *V* = *ab* ^2^ */*2, where *a* and *b* are tumor length and width, respectively. Tumor growth inhibition (TGI,  %) formula is (TuGcontrol−TuGtest)/TuGcontrol × 100 %, where TuG = final tumor size-pretreatment tumor size.

### Determination of intratumoral erlotinib concentration by HPLC

Erlotinib levels in homogenized tumor tissues were determined by reverse-phase high-performance liquid chromatography (HPLC) with UV detection at 345 nm. Separation was achieved on a Waters Symmetry C18 column (150 × 4.6 mm, 5.0 μm; Waters, Milford, MA) preceded by the use of a Symmetry C18 Guard column (3.9 × 20 mm). The mobile phase was 50 mM potassium phosphate buffer (pH 4.8) containing 0.2 % triethylamine and acetonitrile (60:40, v/v), with 1.0 mL/min flow rate at 25 °C. Sample pretreatment involved mixing 500 μL of tumor tissue homogenate with 80 μL of internal standard (70 μg/mL of midazolam in methanol) and 5 mL of tert-butyl methyl ether for 10 min. After centrifugation (650 g, 10 min, 4 °C), the organic top layer was transferred to a clean tube and dried under nitrogen gas at 37 °C. The residue was dissolved in 250 μL of mobile phase. The solution was centrifuged (4,000 g, 30 min) and the supernatant was passed through a microporous membrane filter (Millex-GV 0.22-μm filters, Millipore Corp., Bedford, MA). Insoluble materials were removed by filtration, and the filtrate was analyzed by high-performance liquid chromatography. The calibration curves were linear over a concentration range of 20–4,000 ng/mL (*r*2 > 0.998).

### Statistical analysis

Quantitative data are presented as the mean ± SEM. The Student’s *t* test and/or Mann–Whitney *U* test were used for comparison of two groups and one-way analysis of variance (ANOVA) test was for more than three groups. *P* < 0.05 was considered statistically significant. All data were representative of three independent experiments.

## Results

### Effects of erlotinib/bevacizumab on NSCLC cell lines in vitro

**Fig. 1:**
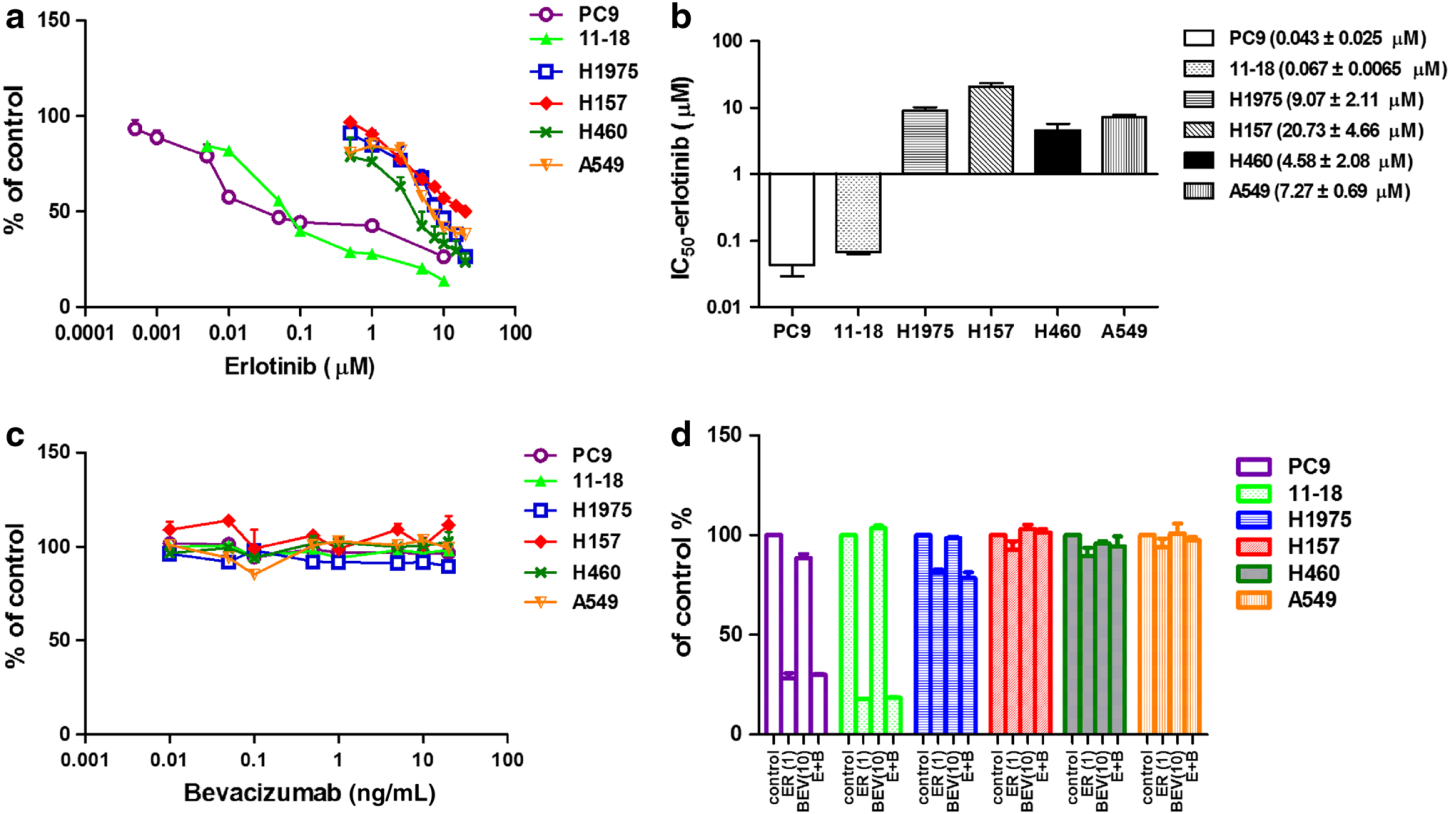
Effects of erlotinib/bevacizumab on NSCLC cell lines in vitro. MTS assay was used to evaluate the effects of erlotinib (**a**), bevacizumab (**c**), and combination of erlotinib and bevacizumab (**d**) on the growth of NSCLC cell lines, which included cell lines with EGFR mutations: PC9 (EGFR exon 19 deletion), 11–18 (EGFR L858), H1975 (EGFR L858R and T790 M mutations) and EGFR wild-type cell lines: H157, H460 and A549. Cells were treated with erlotinib (0–20 µmol/L), bevacizumab (0–20 ng/mL) or combination of these agents (ER 1 µmol/L; BEV 10 ng/mL) for 72 h. The percentage of viable cells is shown relative to that of the untreated control. **b** The IC_50_ of erlotinib in the different cell lines. **d** No significant differences were noted between erlotinib alone and combination treatment in vitro (*P* > 0.05). Results are presented as the mean ± SEM. *ER* erlotinib, *BEV* bevacizumab

We examined the sensitivity of various NSCLC cells to erlotinib in vitro (Fig. 1a). The PC9 (EGFR exon 19 deletion) and 11–18 (EGFR L858) cells were sensitive to erlotinib, with IC_50_ values of 0.043 ± 0.025 µmol/L and 0.067 ± 0.0065 µmol/L, respectively (Fig. 1b). The H1975 cell (L858R + T790 M) and the EGFR wild-type cells (H157, H460 and A549) were resistant to erlotinib, with IC_50_ values of 9.07 ± 2.11, 20.73 ± 4.66, 4.58 ± 2.08, and 7.27 ± 0.69 µmol/L, respectively (Fig. 1b). The differences in sensitivity to erlotinib between the sensitive and the resistant cells were significant (*P* < 0.05).

Next, we applied bevacizumab alone or plus erlotinib to the NSCLC cells in vitro. As reported previously [21], bevacizumab alone did not inhibit the growth of the tested NSCLC cells in vitro (Fig. 1c). Growth inhibition with bevacizumab (10 ng/mL) plus erlotinib (1 µmol/L) was similar to that with erlotinib alone (1 µmol/L; Fig. 1d) in the six NSCLC cells (*P* > 0.05).

### Human VEGF protein expression in NSCLC cell lines

**Fig. 2:**
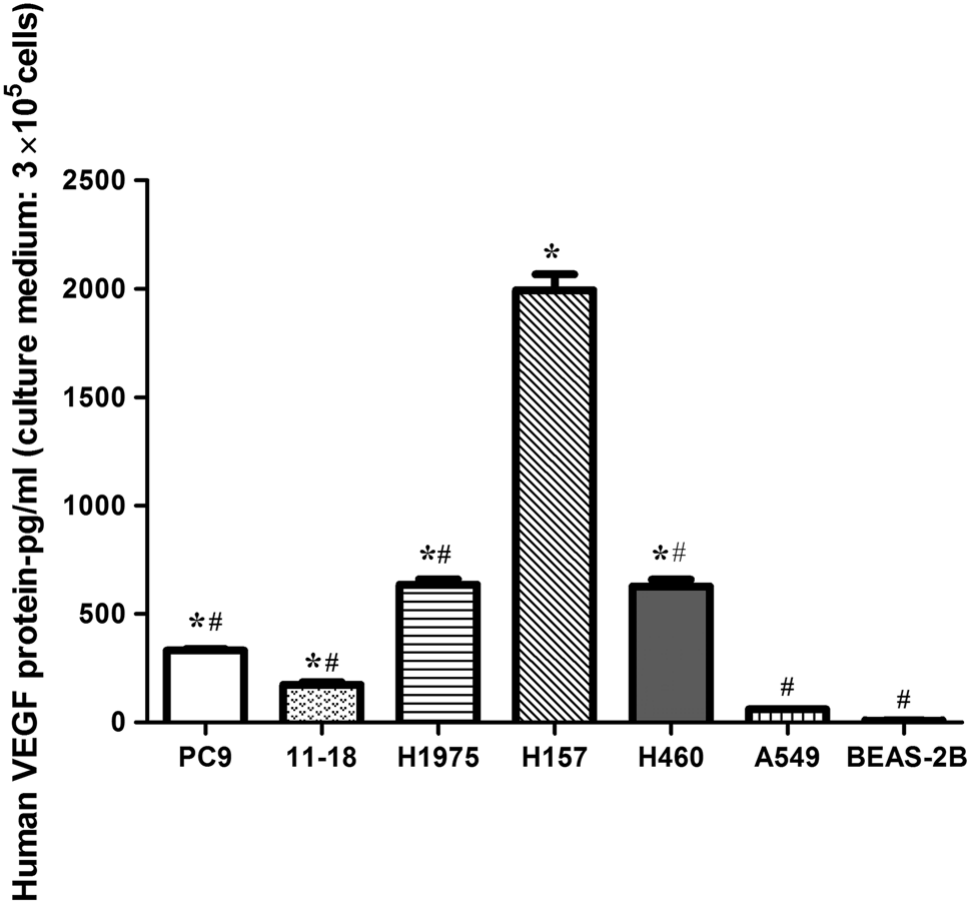
Levels of human VEGF protein in NSCLC cell lines. Human VEGF protein in culture medium (2 mL with free fetal bovine serum) of NSCLC cell lines (3 × 10^5^ cells) and the human bronchial epithelial cell line BEAS-2B (control) was assessed by ELISA. Data are presented as the mean ± SEM. *P < 0.05 for cells compared with BEAS-2B; ^#^ *P* < 0.05 for cells compared with H157

As shown in Fig. 2, VEGF protein expression varied among the cells. In the erlotinib-resistant NSCLC cells, A549 cells secreted the lowest level of VEGF protein into the culture medium, H1975 and H460 cells expressed moderate levels, and H157 cells secreted the highest level. The levels of VEGF protein in erlotinib-sensitive NSCLC cells PC9 and 11–18 cells were lower than those secreted by H1975, H460 and H157 cells. Statistical analysis showed significant differences between the NSCLC cells (except A549) and the control cell BEAS-2B (*P* < 0.05).

### Effects of bevacizumab monotherapy in NSCLC xenograft models

**Fig. 3:**
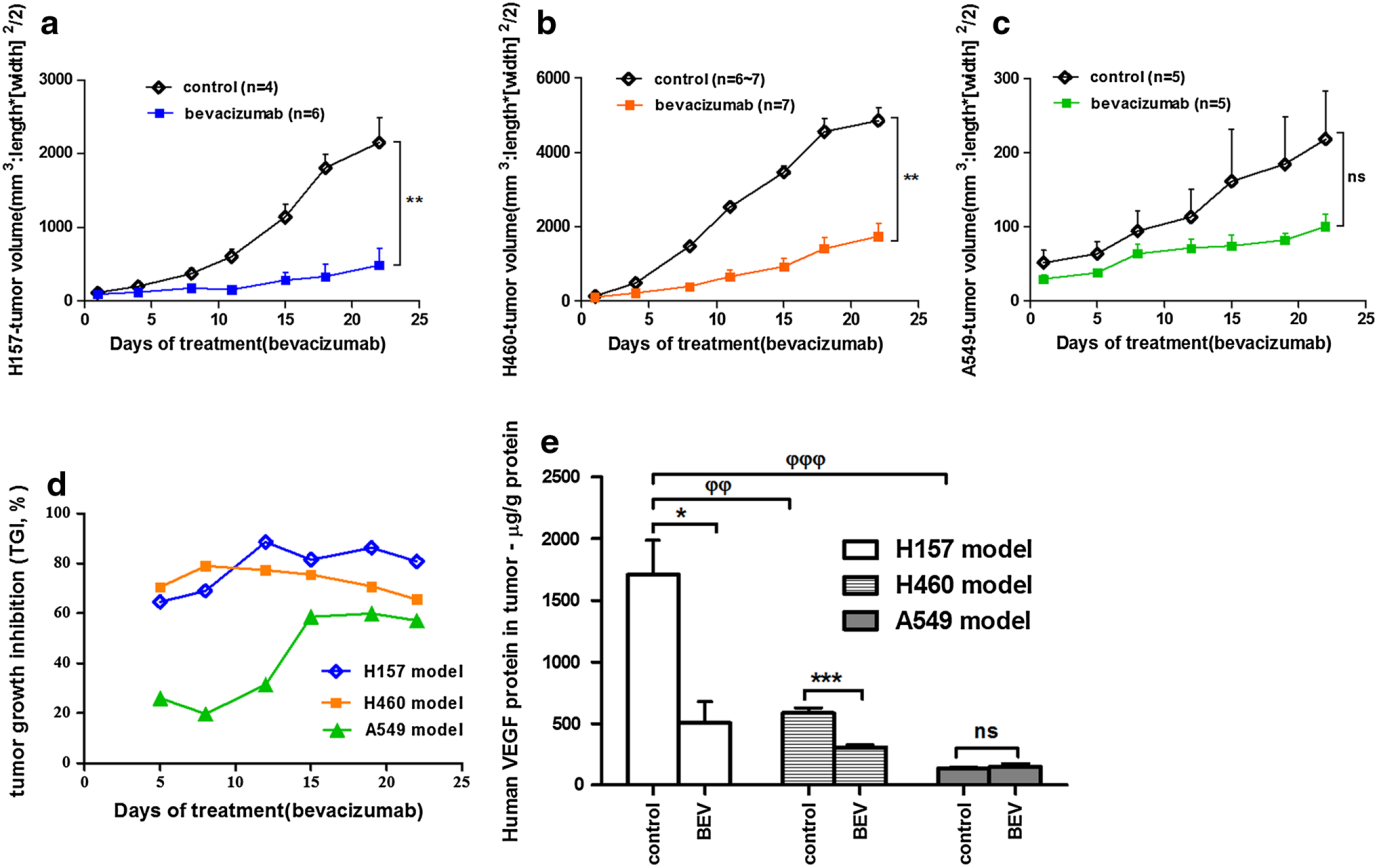
Effects of bevacizumab monotherapy on NSCLC xenograft models with primary resistance to erlotinib. **a, b** and **c** Tumor volume over time in response to bevacizumab (5 mg/kg; *n* = 4–7). The Mann–Whitney *U* test was used to compare tumor volume at the last measurement between the groups (ΔT/ΔC): **P < 0.01 in H157 tumors (**a**) and H460 tumors (**b**); ^ns^ *P* > 0.05 in A549 tumors (**c**). **d** TGI  % in each model was calculated from the beginning of bevacizumab treatment. **e** The levels of human VEGF protein in tumor tissues of H157, H460 and A549 models were assayed by ELISA. The Student’s *t* test was used to compare bevacizumab and vehicle treatment in each model: *P < 0.05, ***P < 0.001, ^ns^ *P* > 0.05. For comparison between the three xenograft tumors, one-way ANOVA was used: ^φφ^ *P* < 0.01, ^φφφ^ *P* < 0.001. Data are expressed as the mean ± SEM. *ER* erlotinib, *BEV* bevacizumab

According to the results in vitro, EGFR wild-type cells H157, H460 and A549 are erlotinib-resistant cells that express high, moderate and low levels of VEGF protein, respectively. We assessed the effect of bevacizumab monotherapy in these three xenograft models. Bevacizumab (5 mg/kg) was well tolerated with no significant effects on body weight (Supplementary Fig. 1) [22] and showed significant antitumor activity in the H157 and H460 models (*P* < 0.01; Fig. 3a, b) rather than in the A549 model (Fig. 3c). TGI  % were 80.82, 65.62 and 57.14 % in H157, H460 and A549 models at the end of treatment, respectively (Fig. 3d). These results suggested the NSCLC xenograft model that expressed high levels of VEGF protein was more sensitive to VEGF blockade than the models with lower VEGF protein.

We also examined the levels of human VEGF protein in tumor tissues. Consistent with the previous observations in vitro, the level of VEGF protein in the H157 tumor tissue was highest, followed in order by H460 and A549 tumor tissues (*P* < 0.01; Fig. 3e). Bevacizumab significantly reduced the level of VEGF protein in tumor tissues from the H157 (*P* < 0.05) and H460 (*P* < 0.001) models but not in that from the A549 model (*P* > 0.05). This result was due to the function of bevacizumab that neutralizes VEGF [23, 24]. The changes in VEGF levels observed between the bevacizumab and control groups also reflected the sensitivity of NSCLC xenografts to bevacizumab treatment.

### Antitumor activity of erlotinib combined with bevacizumab in NSCLC xenograft models

Current chemotherapy/targeted regimens have used multiple agents for the treatment of carcinomas to improve efficacy and avoid the development of resistance [8, 25, 26]. Because the effect of combined agents to erlotinib is difficult to detect in erlotinib-sensitive tumor, we investigated the efficacy of erlotinib plus bevacizumab in erlotinib-resistant xenografts in a separate experiment.

**Fig. 4:**
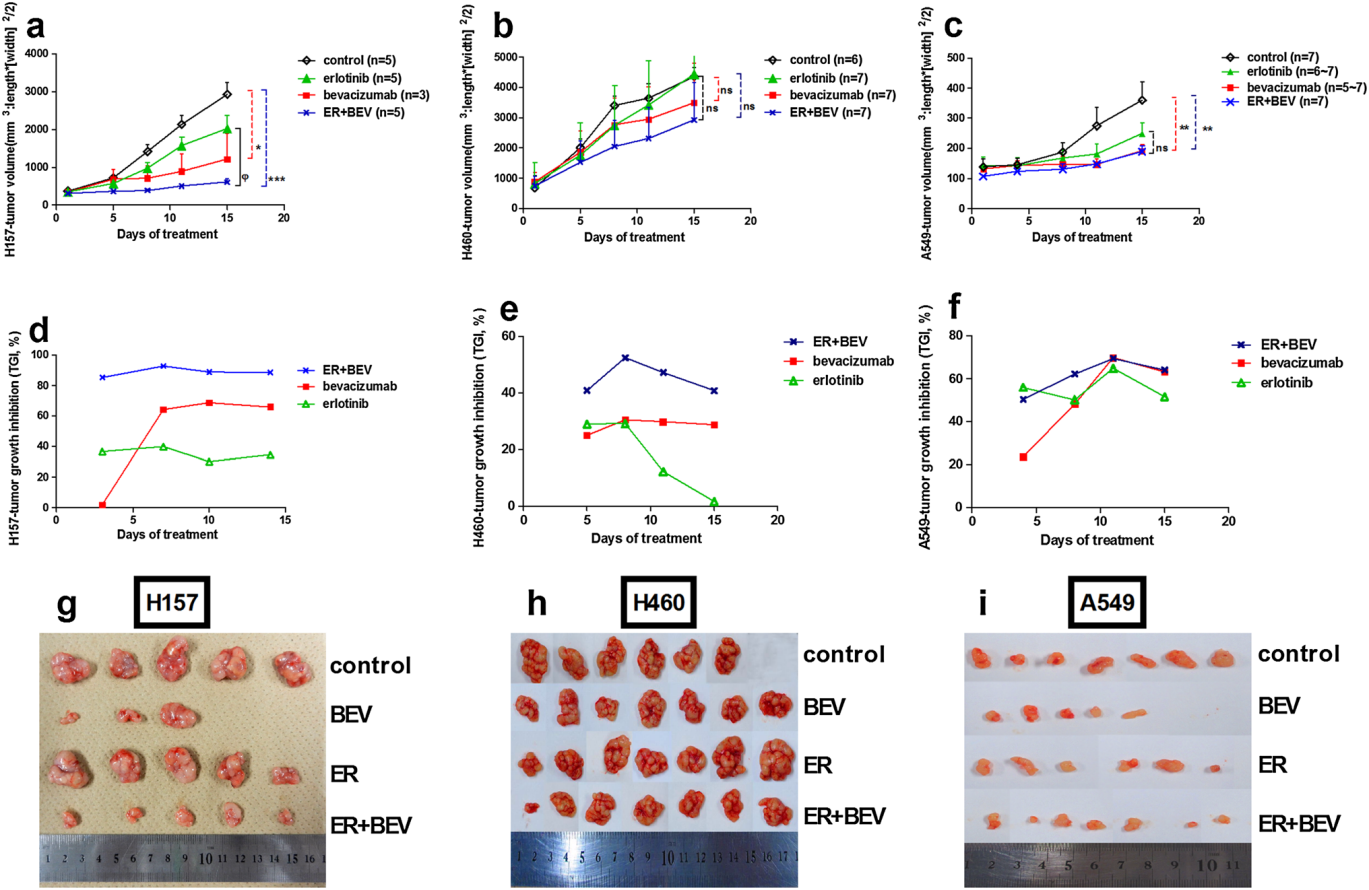
Effects of erlotinib plus bevacizumab on tumor growth in NSCLC xenograft models. **a, b** and **c** Changes in tumor volume over time in response to treatment with vehicle, bevacizumab (5 mg/kg), erlotinib (100 mg/kg) or combination of bevacizumab (5 mg/kg) and erlotinib (100 mg/kg) for 2 weeks (*n* = 3–7). One-way ANOVA was used to compare tumor volume at the last measurement between the treatment groups in each xenograft model: H157 model (**a**), H460 model (**b**), and A549 model (**c**). ***P < 0.001, *P < 0.05, **P < 0.01 for combination treatment or bevacizumab treatment compared with the vehicle (ΔT/ΔC); ^φ^ *P* < 0.05 for combination treatment compared with erlotinib alone (ΔT/ΔT′); and ^ns^ *P* > 0.05 means no significant differences. **d, e** and **f** TGI  % by erlotinib, bevacizumab and combination treatment in three models. **g, h** and **i** Images of tumor samples in three xenografts. Data are expressed as the mean ± SEM. *ER* erlotinib, *BEV* bevacizumab

Erlotinib alone did not cause significant inhibition of tumor growth compared with vehicle in the H157 model (TGI < 40 %; Fig. 4a, d) or in the H460 model (TGI < 30 %; Fig. 4b, e). Erlotinib plus bevacizumab achieved significant tumor inhibition compared with treatment with erlotinib alone (*P* < 0.05) or vehicle (*P* < 0.001) in the H157 model (TGI > 85 %; Fig. 4d and Supplementary Fig. 2a). In contrast, combination treatment inhibited H460 tumor growth by about 40 % by the end of study (Fig. 4e), but the inhibition was not significantly greater than that of erlotinib alone or vehicle (Fig. 4b and Supplementary Fig. 2b). Although A549 cells were resistant to erlotinib in vitro, A549 tumor growth in nude mice was moderately suppressed by erlotinib (TGI > 52 %; Fig. 4c, f). Blockade by combined treatment inhibited A549 tumor growth more than treatment with vehicle (*P* < 0.01), but was not more effective than treatment with either agent alone (Fig. 4c and Supplementary Fig. 2c).) No substantial weight loss was observed during treatment (Supplementary Fig. 3). Taken together, these results indicated that erlotinib plus bevacizumab was capable of inhibiting tumor growth and/or partially reversing resistance to erlotinib in established xenografts with high VEGF expression.

### Concentration of erlotinib in tumor tissues of xenograft models

Previous studies have shown that bevacizumab can enhance drug delivery to tumors [15]; however, this remains controversial [16, 17]. According to a previous study [27], the erlotinib concentration in mouse tumors reaches its peak concentration within 1 h after p.o. administration and declines rapidly for the next 6 h. Therefore, we excised tumor samples in athymic mice 1 h after administrating erlotinib p.o. on the last day of treatment and observed the changes in intratumoral erlotinib concentration.

**Fig. 5:**
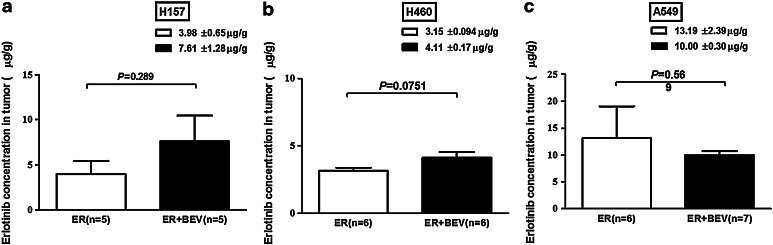
Erlotinib concentration in tumor tissues. HPLC was used to determine the erlotinib concentration in the tumor tissues from the xenograft models treated with erlotinib alone or combined with bevacizumab. Data are expressed as the mean ± SEM. The Student’s *t* test was used to compare erlotinib and combination groups in each model: *P* = 0.289 (**a** H157 model), *P* = 0.0751 (**b** H460 model) and *P* = 0.569 (**c** A549 model). *ER* erlotinib, *BEV* bevacizumab

Erlotinib concentrations in the H157, H460 and A549 tumor tissues treated with erlotinib alone or plus bevacizumab reached 3.98 ± 0.65 and 7.61 ± 1.28 µg/g (*P* = 0.289; Fig. 5a); 3.15 ± 0.094 and 4.11 ± 0.17 µg/g (*P* = 0.0751; Fig. 5b); 13.19 ± 2.39 and 10.00 ± 0/0.30 µg/g (*P* = 0.569; Fig. 5c), respectively. The changes in intratumoral erlotinib concentration were consistent with the antitumor activity of erlotinib plus bevacizumab treatment in these three xenografts. Compared with an increased erlotinib concentration following combination treatment in H157 tumor tissue, A549 tumors showed the opposite results.

## Discussion

Erlotinib monotherapy is approved for treatment of patients with advanced or metastatic NSCLC, while bevacizumab monotherapy is not standard for NSCLC treatment [5, 7]. The objective of our study was to determine whether there were conditions under which the addition of bevacizumab would enhance antitumor activity of erlotinib against NSCLC tumors in vitro and in vivo. First, we found that erlotinib plus bevacizumab were no extra inhibitory than erlotinib alone in vitro. This result is consistent with those of a previous study [21] and may be explained by the fact that VEGFR is expressed on vascular endothelium but not on malignant cells in human solid tumor types (including lung cancer) [28]. Because the change of erlotinib concentration is undetectable in erlotinib-sensitive tumor due to effective apoptosis induction to the cancer cells with low level of erlotinib, we then produced xenograft models bearing primarily erlotinib-resistant NSCLC cells to investigate the erlotinib accumulation in the tumor with bevacizumab combination. We found that the tumors expressing higher levels of VEGF protein were more responsive to inhibition by bevacizumab and combination treatment. These results suggest that the effect of dual inhibition of EGFR and VEGF is dependent on VEGF expression in EGFR TKI-resistant xenografts. Finally, we demonstrated a trend toward increasing concentrations of erlotinib in the tumor tissues during treatment with erlotinib and bevacizumab compared with erlotinib alone. This effect also appeared to be influenced by the levels of VEGF protein.

VEGF plays a central role in angiogenesis and is necessary for endothelial cell survival in tumors, while the expression of VEGF protein depends on the type of cancer [29]. Decreasing VEGF levels by bevacizumab is known to block angiogenesis, transiently normalize tumor vessels, sensitize tumors to radiotherapy and chemotherapy, improve tumor oxygenation and decrease interstitial fluid pressure, as well as restore delivery of drugs into the tumor [15, 30]. These might explain why the combination treatment worked better in the H157 model with high VEGF expression. In addition, because EGFR plays a vital role in the regulation of cell proliferation, survival and differentiation [31], partial normalization of tumor vessels by bevacizumab could cause proliferation of the tumor cells, which could make them more sensitive to EGFR TKI. As reported previously, EGFR TKI is known to be dose-related inhibition of EGFR function [27, 32, 33]. Therefore, bevacizumab combined with erlotinib is reasonable to enhance the antitumor effect by increasing intratumoral concentration of erlotinib.

On the other hand, the differences in efficacy for combinations of erlotinib plus bevacizumab between our study and the clinical trials [12, 13] are just like previous reports, that clinical efficacy is lower than that observed in preclinical cancer models [15, 16, 31, 34]. One possible explanation is that the efficacy of bevacizumab alone or combined with other agents differs among tumor types, such as transplantable tumors in mice, spontaneous tumors and tumors from patients, which exhibit different degrees of vessel abnormality and levels of VEGF protein [15]. An alternate explanation for the variability in treatment efficacy is that vessel normalization by VEGF blockade is limited to a small window in treatment time. The window of normalization in murine models is relatively short and occurs soon after administration of bevacizumab compared with that in humans [29], which may explain several paradoxical findings reported recently and suggest that the schedule and dosing of combination therapies warrant considerable attention. For example, bevacizumab and cetuximab (anti-EGFR monoclonal antibody) have shown promising results in clinical trials in NSCLC [35]. However, this is at odds with the results of a study which showed that bevacizumab reduced tumor uptake of cetuximab in SUM149 xenografts (a breast cancer xenograft) [16]. A bevacizumab/docetaxel combination was more effective than docetaxel alone in reducing breast and prostate cancer cell growth [36], but a rapid decrease in the delivery of docetaxel to tumors after bevacizumab therapy was observed in another study [17]. Notably, although the erlotinib concentration in the combination group of H157 model with high VEGF levels was much higher than that in the erlotinib only group, no significant difference in intratumoral erlotinib concentration was observed between these two groups in any of the three models. This might be attributed to different blood flow even within the same tumor, because abnormal vessels in tumors result in continuous vessel remodeling, as well as facilitate drug distribution in perfused and leaking vessels [16, 17].

Until now, it is controversial whether EGFR TKIs alone or combined with other agents are recommended to patients with EGFR wild-type NSCLC as a second- or third-line treatment [37–42]. Lung cancer is not homogenous, and any change in histology or mutational status could happen after chemotherapy and/or targeted therapy, which suggests that EGFR wild-type should not be invalid indication for EGFR TKI. Although cytotoxic chemotherapy remains the backbone of therapy for patients with advanced NSCLC, EGFR TKIs and VEGF inhibitors are potential ones. Therefore, further understanding of mechanisms and modes in dual inhibition of EGFR and VEGF is a priority. As there are inconsistent reports on VEGF blockade affecting the delivery of combined drugs, the precise effects of these reactions should be further investigated.

In conclusion, we demonstrated that bevacizumab may be useful for enhancing antitumor activity of erlotinib by increasing the intratumoral concentration of erlotinib in some tumors that express high levels of VEGF protein. Our study is limited by the small number of tissue samples evaluated and not assessing other angiogenic factors (such as basic FGF or PDGF [38] ) that tumor may depend on. Besides, to establish histologically and genetically accurate models of human cancer, genetically engineered model will be used for further study. Overall, it is important to understand the principles and mechanisms of the combination approach for effectively translating preclinical studies into clinical practice for better efficacy.

## Electronic supplementary material

Below is the link to the electronic supplementary material.

Supplementary material 1 (PDF 135 kb)

Supplementary material 2 (DOCX 13 kb)
