# Supplementary figures and images for: Addition of bevacizumab enhances antitumor activity of erlotinib against non-small cell lung cancer xenografts depending on VEGF expression
Source: Cancer Chemother Pharmacol. 2014 Oct 26;74(6):1297–305. doi: 10.1007/s00280-014-2610-x (PMC4236614; doi:10.1007/s00280-014-2610-x)

# Supplementary Fig. 1

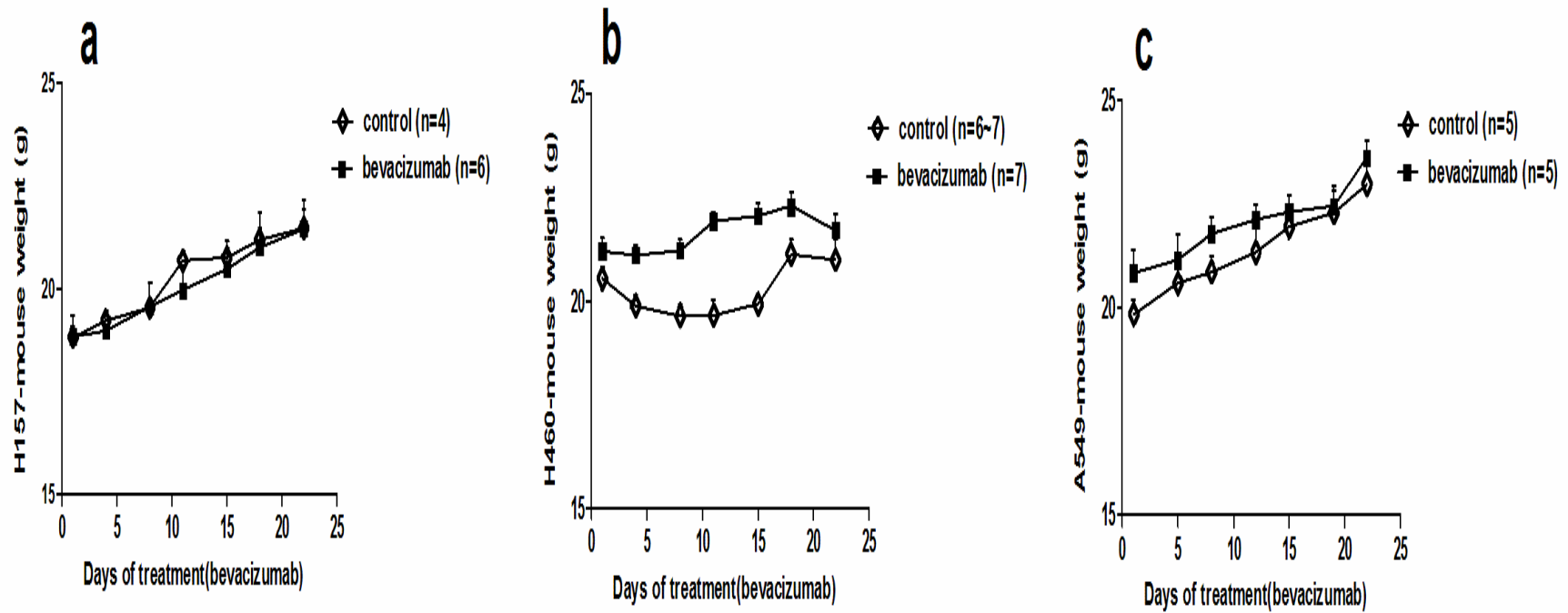

## Supplementary Fig. 2

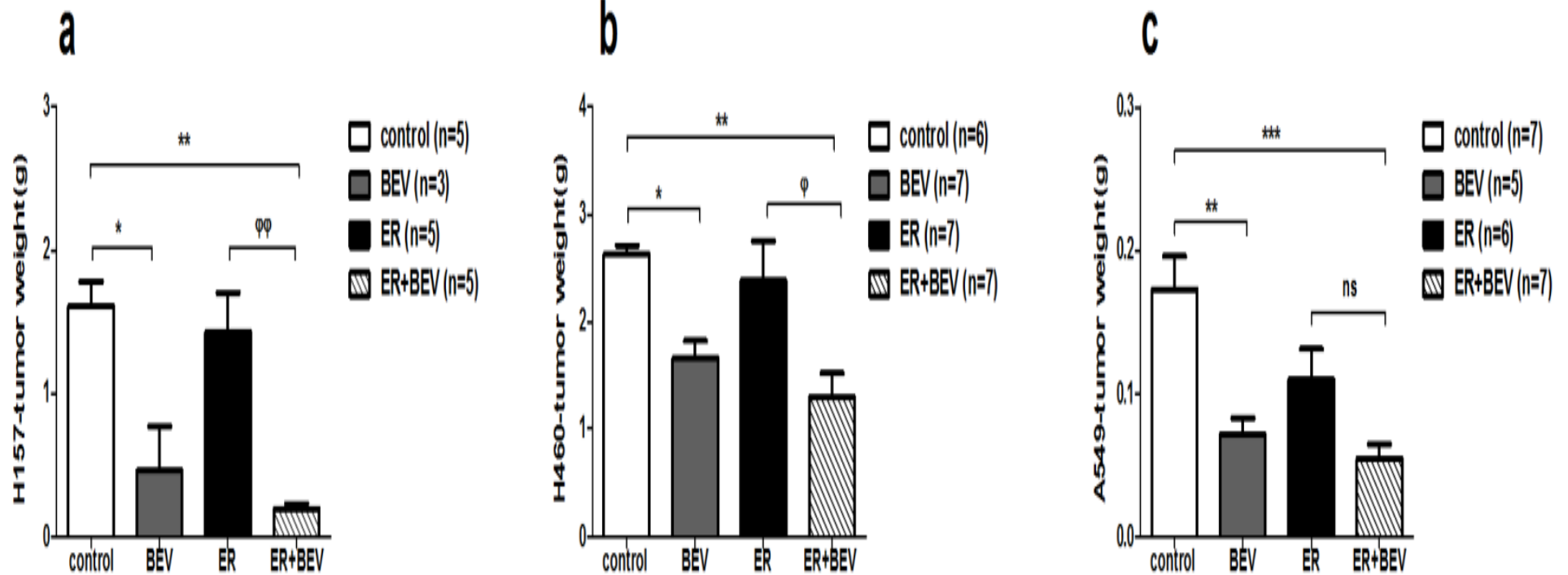

## Supplementary Fig. 3

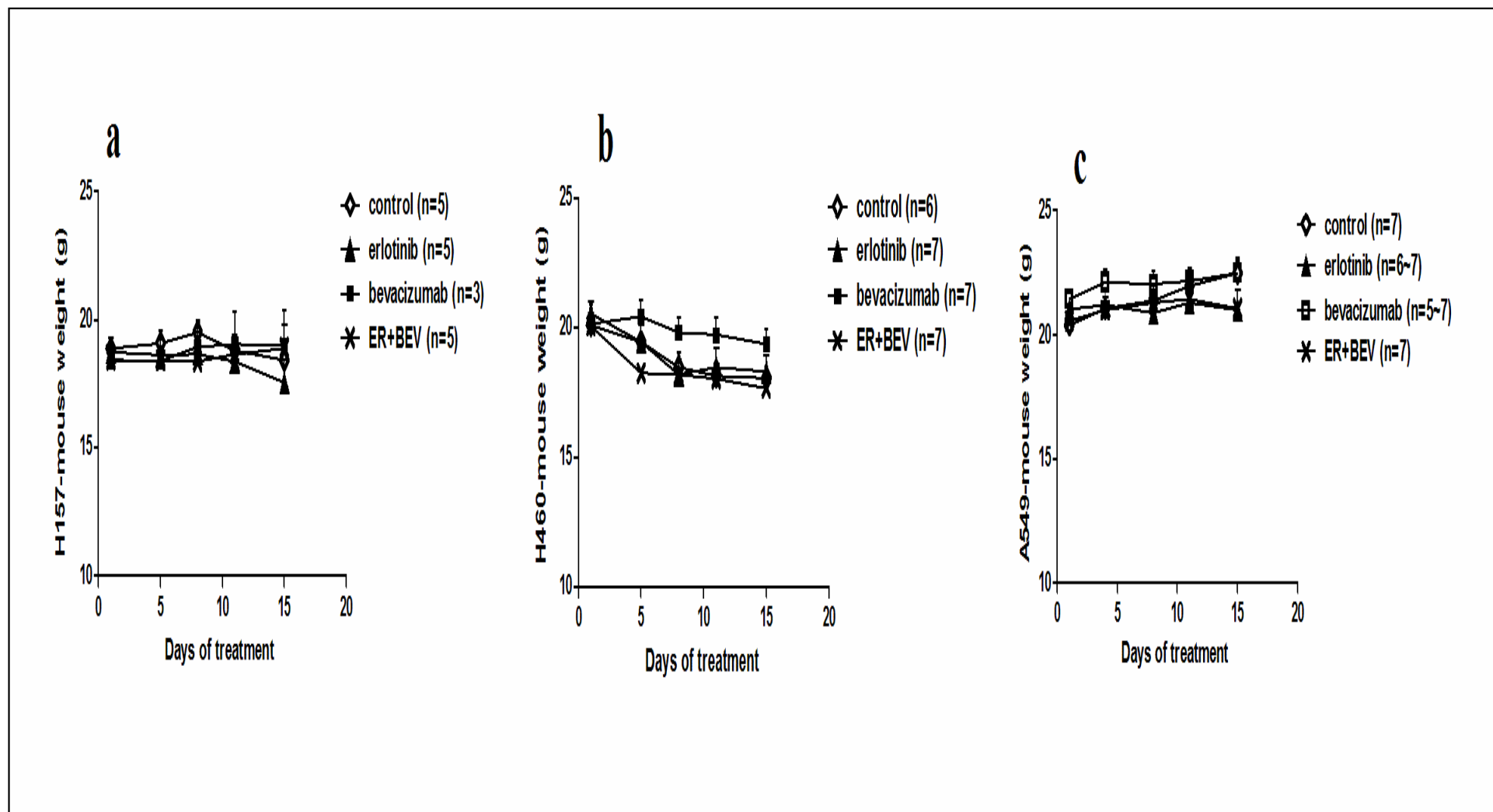

Supplement: Supplementary file 1 — Supplementary material 1 (PDF 135 kb) [file 280_2014_2610_MOESM1_ESM.pdf]
