# Supplementary material for: Addition of bevacizumab enhances antitumor activity of erlotinib against non-small cell lung cancer xenografts depending on VEGF expression
Source: Cancer Chemother Pharmacol. 2014 Oct 26;74(6):1297–305. doi: 10.1007/s00280-014-2610-x (PMC4236614; doi:10.1007/s00280-014-2610-x)
Supplement: Supplementary file 2 — Supplementary material 2 (DOCX 13 kb) [file 280_2014_2610_MOESM2_ESM.docx]

**Supplementary Figure Legends**

**Supplementary Fig. 1.** Body weight of xenograft models treated with bevacizumab monotherapy. No significant effect on body weight (≥ 20 % of body weight at the start of treatment) was observed in H157 (**a**), H460 (**b**), or A549 (**c**) models as a result of bevacizumab treatment (5 mg/kg/twice weekly).

**Supplementary Fig. 2. Weight of tumor samples in three xenografts.** The weights of tumor tissue are shown in **a** (H157 tumors), **b** (H460 tumors) and **c** (A549 tumors). One-way ANOVA was used to compare the differences between the treatment groups in each model: ***P*< 0.01, **P*< 0.05, ^φφ^*P*< 0.01, ^φ^*P*< 0.05, and ^ns^*P*> 0.05. Data are expressed as the mean ± SEM. ER, erlotinib; BEV, bevacizumab.

**Supplementary Fig. 3.** Body weight of H157 (**a**), H460 (**b**), and A549 (**c**) xenograft models treated with vehicle, bevacizumab (5 mg/kg/twice weekly), erlotinib (100 mg/kg/day), or a combination of the two agents. No significant effect of treatment on body weight (≥ 20 % of body weight at start of treatment) was observed. ER, erlotinib; BEV, bevacizumab.
